# Non-destructive comparative evaluation of fossil amber using terahertz time-domain spectroscopy

**DOI:** 10.1371/journal.pone.0262983

**Published:** 2022-03-30

**Authors:** Phillip Barden, Christine E. Sosiak, Jonpierre Grajales, John Hawkins, Louis Rizzo, Alexander Clark, Samuel Gatley, Ian Gatley, John Federici

**Affiliations:** 1 Federated Department of Biological Sciences, New Jersey Institute of Technology, Newark, New Jersey, United States of America; 2 Division of Invertebrate Zoology, American Museum of Natural History, New York, New York, United States of America; 3 Department of Physics, New Jersey Institute of Technology, Newark, New Jersey, United States of America; Oregon State University, UNITED STATES

## Abstract

Fossilized plant resins, or ambers, offer a unique paleontological window into the history of life. A natural polymer, amber can preserve aspects of ancient environments, including whole organisms, for tens or even hundreds of millions of years. While most amber research involves imaging with visual light, other spectra are increasingly used to characterize both organismal inclusions as well as amber matrix. Terahertz (THz) radiation, which occupies the electromagnetic band between microwave and infrared light wavelengths, is non-ionizing and frequently used in polymer spectroscopy. Here, we evaluate the utility of amber terahertz spectroscopy in a comparative setting for the first time by analyzing the terahertz optical properties of samples from 10 fossil deposits ranging in age from the Miocene to the Early Cretaceous. We recover no clear relationships between amber age or botanical source and terahertz permittivity; however, we do find apparent deposit-specific permittivity among transparent amber samples. By comparing the suitability of multiple permittivity models across sample data we find that models with a distribution of dielectric relaxation times best describe the spectral permittivity of amber. We also demonstrate a process for imaging amber inclusions using terahertz transmission and find that terahertz spectroscopy can be used to identify some synthetic amber forgeries.

## Introduction

Amber represents a data-rich window into the ancient evolutionary and environmental history of the planet. Amber derives from resin, a liquid substance exuded from plants most frequently following wounds or disease. Resin is composed primarily of terpenoid compounds, which act as defensive agents against insects, herbivores, fungi, or microbes as well as after physical damage or during some climatic events [1–7]. The exact composition of resin is dependent on the source plant [8,9]. Once exposed to light and air, the terpenoid compounds in amber begin to polymerize; as resin is buried in sedimentary layers, heat and pressure continue the polymerization process. Over millions of years, this chemical transformation results in amber: older ambers have greater degrees of polymerization, but polymerization may also be influenced by exposure to heat and pressure [3,10]. This polymerization process effectively seals any organic or inorganic material trapped inside of the original resin exude. In the amber research community, the term ‘inclusion’ refers to any material, void, or crack which is trapped inside of the polymerized resin.

There are over one hundred known amber deposits ranging in age from just a few million years ago to approximately 320 Ma (Megaannum) [9,11] with the oldest biological inclusion-yielding amber dated to the Triassic ca 230 Ma [12]. Inclusions entombed within amber are not mineralized replacements as in other fossilization modes, and the high fidelity of amber preservation allows for detailed study of ancient organisms, especially arthropods [13]. Amber matrix itself provides important paleobotanical information relating to ancient climate and environments, for example by demonstrating that present-day Europe was subtropical in the Eocene ~40 million years ago [14]. Importantly, it is possible to identify botanical sources responsible for some amber deposits through chemical analyses and fossilized plant associations such as bark [15]. While amber has been appreciated for millennia, the application of technologies such as X-ray based micro-CT [16–18] and spectroscopy [14] have accelerated discovery in the study of inclusions and amber chemistry, respectively. Even as light microscopy, X-ray imaging, Raman spectroscopy, and infrared spectroscopy [19] remain the most common methods in amber research, the terahertz (THz) spectral range has recently been developed as an imaging technique and has been used in polymer spectroscopy [20].

Terahertz wavelengths (3mm to 100 μm) exist between microwave and infrared light. Terahertz radiation may be used in non-destructive imaging of low absorbance materials because it is non-ionizing. Only recently has terahertz imaging and spectroscopy been applied to archeological and paleontological applications [21–23]. Applications include non-invasive analysis of artworks [24], non-destructive evaluation of 14^th^ to 18^th^ century wax seals [25], and non-destructive imaging of mummies. Some advantages of terahertz imaging for human remains [26] include better depth resolution compared to conventional X-ray based CT scans, comparable depth resolution to micro-CT scans, and the capability to image complete bodies that may not fit into conventional micro-CT scanners. In addition to imaging capabilities, terahertz techniques enable spectroscopic identification of materials of archeological importance. As an example, embalming materials contained in bandages have been identified based on terahertz time-domain spectroscopic analysis [27].

There are few publications related to characterization of amber in the terahertz spectral range. One research paper [28], centers around characterizing a single deposit of Baltic amber. In that work, the spectral transmission through amber is measured from 0.14–6.5 THz.

Perhaps the greatest bottleneck in amber-based paleontology is preparation time. Upon initial collection, rough amber specimens are typically surrounded by entirely opaque matrix that must be removed to screen for scientifically valuable inclusions such as organisms, minerals, or even atmospheric gas. Almost always, trimming is done without prior visual screening; inclusions may be damaged in the process. Even with dedicated technicians, it can take years to screen through multiple kilograms of amber. Moreover, some amber is opaque or turbid throughout, and so it is not possible to locate inclusions with visible light microscopy. In the case of New Jersey amber–dated to the Turonian approximately 92 million years ago–as many as 70% of specimens are almost entirely opaque to visible light (D.A. Grimaldi, American Museum of Natural History, Pers comm.). A clear need exists for rapidly identifying inclusions in amber both to reduce preparation time, as well as in cases of high opacity. While other technologies exist for screening (e.g. X-ray imaging and computed tomography), they are time intensive, requiring lengthy setup and tube warm-up. X-ray radiation also may damage specimens or fail to resolve details between matrix and inclusions in rapid scan settings. We report the first terahertz-based images of amber as a proof-of-concept for screening utility. Although this methodology is in early stages, terahertz may be well suited to identifying inclusions in some conditions, particularly as the imaging process is refined and cost of equipment is reduced over time.

Here, we investigate the application and utility of terahertz spectroscopy and imaging in paleontological research. Terahertz spectroscopy is applied to fossil samples ranging from ~16 to 119 Ma from the Dominican Republic, Mexico, the Baltic Sea, India, Arkansas, Wyoming, New Jersey, Myanmar, Spain, and Lebanon. This diversity of deposits represents a wide array of differing chemistries, ages, and proposed source resins, allowing for a comparative assessment of terahertz spectroscopy as it relates to botanical origins and age. We assess alternative models for describing the permittivity of amber and evaluate terahertz as a method for the characterization and rapid identification of inclusions within amber matrix. Finally, we demonstrate that terahertz spectroscopy is suitable for the identification of some fraudulent amber fakes.

## Materials and methods

### Terahertz spectroscopy of amber

Amber deposits exhibit unique chemistry based on age and botanical source [9]. Currently, investigators employ techniques such as FT-Raman spectroscopy, mass spectrometry, solid phase microextraction–gas chromatography–mass spectrometry, and infrared spectroscopy to determine the unique spectral or chemical “fingerprint” of deposits. However, some techniques are not able to discern between deposits [19] and so multiple spectra may be brought to bear on problems such as determining whether or not two geographically distant deposits are in fact the same source and age [29,30]. Can terahertz spectroscopy and imaging provide a new dimension in amber characterization?

Terahertz optical properties of materials such as polymers, plastics, and other dielectric materials are typically modelled using a Debye model in which the permittivity of the material is given by

$$\varepsilon (\omega )=\varepsilon_{\infty }+\frac{\varepsilon_{s}-\varepsilon_{\infty }}{1+i\omega \tau }$$ (1)

where *ω* is the frequency in radians/second, *ε*_*s*_ is the permittivity at zero frequency, *ε*_∞_ is the high frequency limit of the permittivity, and *τ* is the Debye relaxation time. The relaxation time is a measure of the time-lag in the response of the material to a changing electric field. The complex refractive index is related to the permittivity by ${\tilde{n}}^{2}=\varepsilon$. The Debye model is based on a single relaxation time *τ* to describe dielectric relaxation in the material. If instead of a single relaxation time, a distribution of relaxation times better describes dielectric relaxation in a disordered system, the Debye model can be modified using a Cole-Cole, Cole-Davidson, or Havriliak-Negami equation [31]. These various models for dielectric relaxation are given by limiting cases of the Haavriliak-Negami equation below for the real and imaginary part of the permittivity

$$\varepsilon (\omega )=\varepsilon_{r}-i\varepsilon_{i}=\varepsilon_{\infty }+\frac{\varepsilon_{s}-\varepsilon_{\infty }}{{\left[1+{\left(i\omega \tau \right)}^{\sigma_{CC}}\right]}^{\sigma_{CD}}}$$ (2)

where 0<*σ*_*CC*_≤1 and 0<*σ*_*CD*_≤1. For the Cole-Cole model, *σ*_*CD*_ = 1 while the Cole-Davidson model uses *σ*_*CC*_ = 1. For the Debye model of Eq (1), *σ*_*CC*_ = *σ*_*CD*_ = 1 in Eq (2). In the Cole-Cole model, *σ*_*CC*_ is a measure of the interaction between neighboring dielectric dipoles. The Cole-Cole model includes the effect of symmetric broadening of the spectral peak while the Cole-Davidson model accounts for asymmetric broadening.

**Fig 1:**
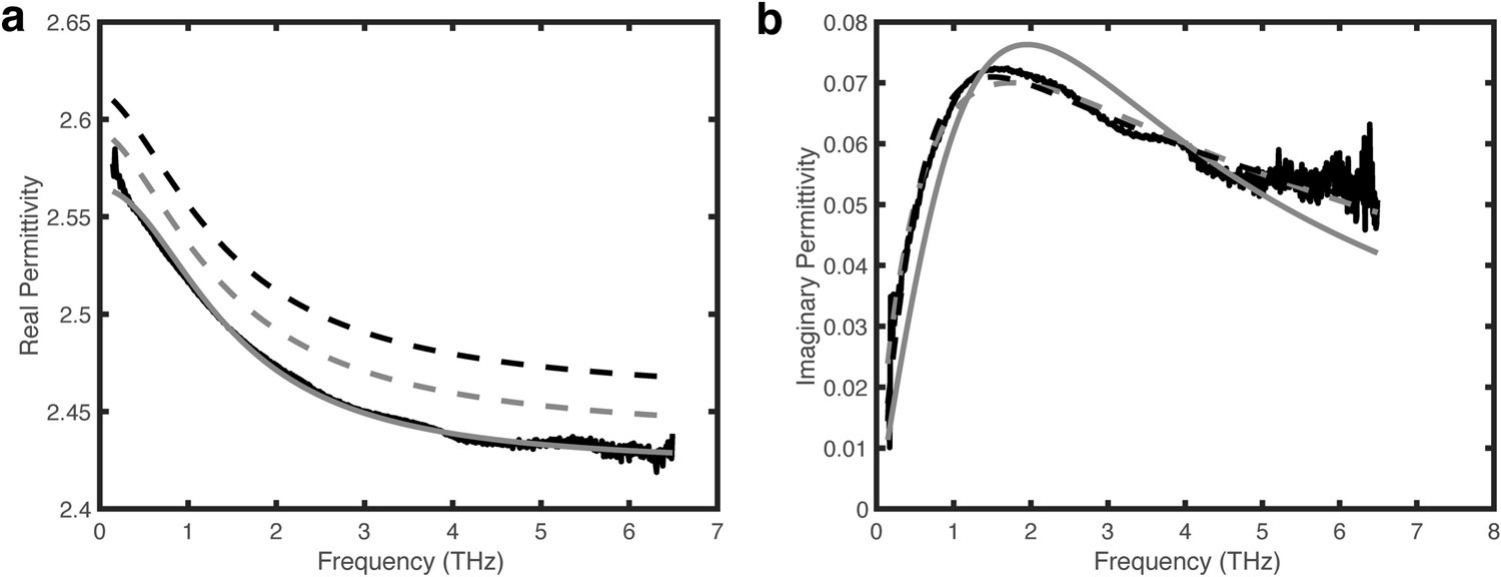
Data of real and imaginary part of permittivity. Data (black line) from Fig 3 of Sasaki et. al. Data at below 0.146 THz has been removed due to the poor signal-to-noise ratio. (a) Fit of real part of permittivity to Debye (solid gray line), Cole-Cole (dashed gray line), and Cole-Davidson (dashed black line) models. For clarity, the Cole-Cole and Cole-Davidson fits have been offset by 0.02 and 0.04, respectively. (b) Fit to imaginary part of permittivity using Debye, Cole-Cole, and Cole-Davidson models.

As a starting point, Fig 1 shows a fit of Sasaki et. al’s [28] spectral data for Baltic Amber to the Debye Model, Cole-Cole, and Cole-Davidson models. While all three models yield good fits to the real permittivity data, note that the imaginary permittivity data is better fit by both the Cole-Cole and Cole-Davidson models compared to the Debye model indicating that the Baltic Amber is better described by a distribution of dielectric relaxation times.

In comparing fits to the Baltic Amber imaginary permittivity data, one must consider not only the R^2^ value of the non-linear least-squares fit, but also the resulting uncertainty (95% confidence level) in the extracted best-fit parameters as well as the residuals. While the Cole-Cole and Cole-Davidson models give relatively high R^2^ values (~0.911 and ~0.938, respectively) for the imaginary part of the permittivity, the uncertainties in the extracted fitting parameters range from 1.1–1.3% for the Cole-Cole model to 1.2–3% for the Cole-Davidson model. The fit to a Debye model yields a significantly lower R^2^ value (~0.46). Of the three different forms of Eq (2), the Cole-Davidson model seems to give the best combination of high R^2^, low uncertainty in the fitted parameters, and a relatively random frequency plot of residuals. Fitting of the imaginary permittivity to the full Haavriliak-Negami equation gives best fit parameters of `*σ*_*CC*_ = 0.975 (ie. small contribution of symmetric broadening) and essentially the same best fit parameters as the Cole-Davidson model (*σ*_*CD*_ = 0.444±0.011, *ε*_*s*_−*ε*_∞_ = 0.215±0.003, *τ* = 0.202±0.006). The applicability of the Cole-Davidson model suggests that dielectric relaxation in Baltic amber results primarily from asymmetric broadening of relaxation times.

### Terahertz instrumentation

For the amber samples characterized in this paper, the samples were imaged and spectroscopically characterized using terahertz time-domain spectroscopy. In this methodology, short time-duration pulses (~ a few picoseconds) of broadbanded (0.1–2 THz) electromagnetic pulses are generated and detected using optoelectronic techniques [32]. Using a T-Ray 5000 terahertz time-domain system from Terametrix, terahertz time-domain waveforms are acquired in either a transmission or reflection mode through the sample for a 160 ps time window. The terahertz time-domain waveforms are analyzed to extract various material parameters including the frequency dependent complex permittivity, transmission, real refractive index, and sample thickness. Images are acquired by scanning the sample through the beam path and acquiring images on a pixel by pixel basis.

Two methods of data analysis are used. For the first method, the transmitted terahertz time-domain waveform is recorded through the sample. A reference waveform is also recorded with the sample removed from the beam path. The Fourier Transform magnitude and phase of the sample waveform are calculated for both the sample and reference. The real refractive index is extracted from the data using the equation

$$n_{r}(\omega )=1+\frac{c_{o}\Delta \phi (\omega )}{\omega L}$$ (3)

in which *c*_*o*_ is the speed of light in a vacuum, *L* is the thickness of the sample, ω is the frequency of the terahertz light in units of radians/sec, and Δ*ϕ*(*ω*) is the difference in the frequency dependent phase of the reference and sample waveforms. Since amber is relatively transparent in the terahertz frequency range, the imaginary refractive index is small compared to the real refractive index. In this situation, one can correct the measured amplitude of the transmitted Fourier transform for Fresnel reflection losses [33] at the air-amber and amber-air interfaces. The imaginary refractive index is calculated using the spectral amplitudes of the transmitted electric fields through the sample and with the sample removed (reference) by

$$n_{i}(\omega )=\frac{c_{o}}{\omega L}\ln(\frac{\left|E_{R}(\omega )\right|}{\left|E_{S}(\omega )\right|}t_{12}t_{21})$$ (4)

with the Fresnel transmission coefficients given by *t*_12_(*ω*) = 2/(1+*n*_*r*_(*ω*)) and *t*_21_(*ω*) = 2*n*_*r*_(*ω*)/(*n*_*r*_(*ω*)+1). The complex permittivity is calculated from the complex refractive index using

$$\varepsilon_{r}-i\varepsilon_{i}={\left(n_{r}+in_{i}\right)}^{2}.$$ (5)

For the above method, the thickness *L* of the sample is typically measured using calipers. If the two polished sides of an amber sample are not exactly parallel, the wedge shape of the sample can lead to non-negligible uncertainty in the thickness *L* and the extracted refractive index and permittivity.

The usable range of spectral data in terahertz time-domain spectroscopy is determined by several factors. At the lower frequency range (<0.2 THz), the spectral power decreases rapidly with decreasing frequency. In addition, the size of the sample relative to the spot size of the focused terahertz beam can play an important role in the introduction of experimental artifacts. As the size of the sample becomes smaller than the spot size of the radiation, some terahertz radiation which passes around the sample can interference with radiation which passes through resulting in artifacts in the spectral signature. In order to minimize this effect for small amber samples, a fixed aperture is placed directly in front of the sample to ensure that only radiation which passes through the sample is detected. At the upper terahertz range, the spectral power decreases logarithmically with increasing frequency. At the upper range of frequencies, the signal-to-noise is limited by the power in the terahertz spectrum and a combination of the sample’s attenuation coefficient and thickness.

**Fig 2:**
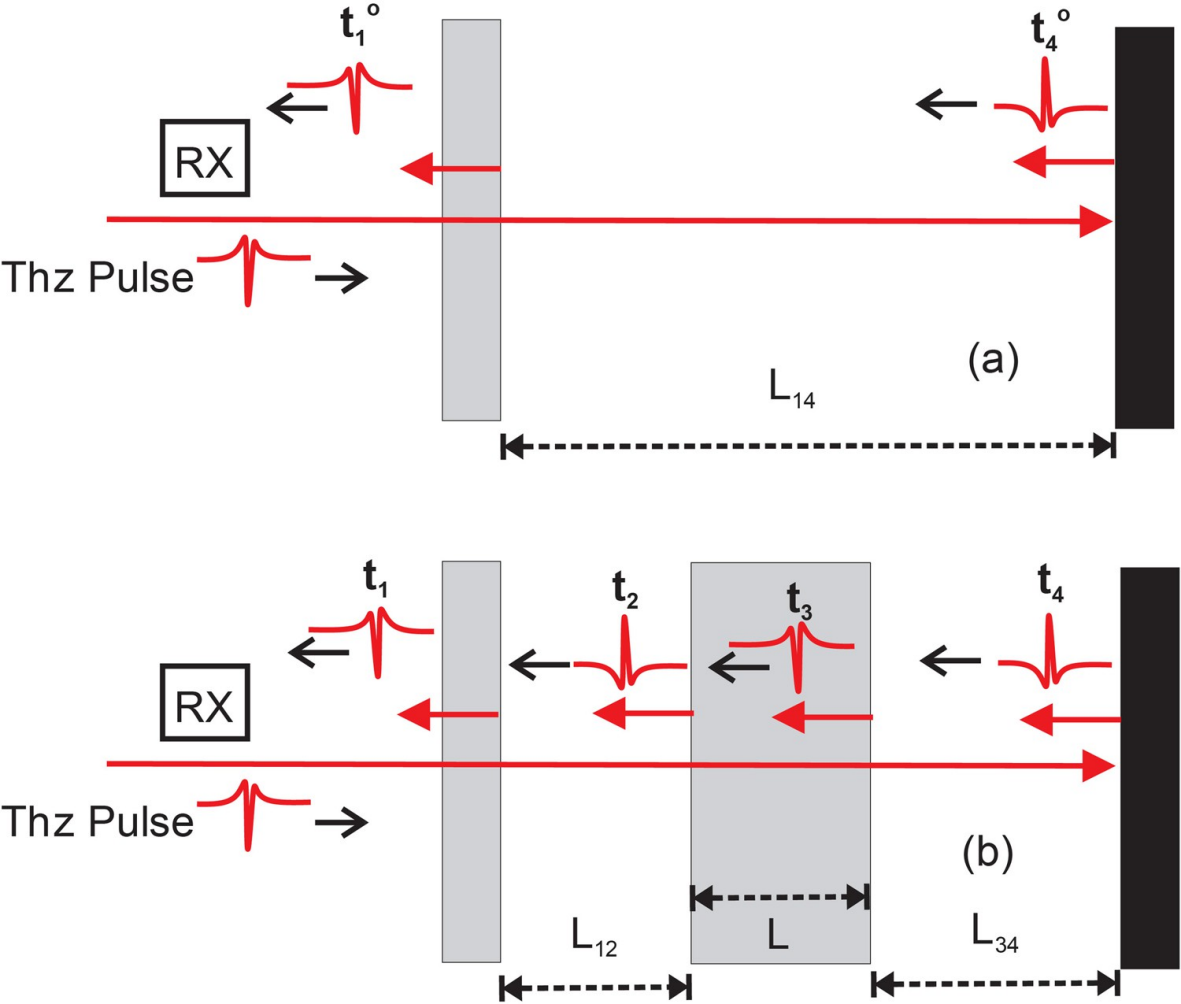
Illustration of the external reference structure for simultaneous determination of sample thickness and refractive index. (a) configuration for determining distance L_*14*_ in the absence of sample. (b) illustration of reflected pulses from back surface of beamsplitter, front surface of sample, back surface of sample, and reflecting mirror.

The second analysis method for extracting the real refractive index and sample thickness simultaneously uses time-domain time of flight analysis in conjunction with an external reference structure (ERS) [34]. As illustrated in **Fig *2***, the ERS consists of a partially reflecting surface (ie. a beamsplitter) and a mirror. When a terahertz pulse interacts with the structure, two main pulses are reflected corresponding to the reflection from the partially reflecting surface and the mirror. By measuring the time-of-flight difference in the arrival time of the two pulses, an accurate measurement of the distance between the reflecting surface and the mirror can be calculated. An accurate arrival time of the two pulses is determined by deconvolving the time-domain waveform with a reference waveform acquired with just the mirror present. Without a sample present, deconvolving the time-domain waveform measures the arrival time of pulses *t*_1_^*o*^ and *t*_4_^*o*^ as illustrated in **Fig *2***A. The distance between the back surface of the beamsplitter and the front surface of the mirror is calculated using simple time-of-flight to be

$$L_{14}=c_{o}(t_{4}{}^{o}-t_{1}{}^{o})/2.$$ (6)

The factor of 2 is present since the pulse which reflects from the mirror transmits through the distance *L*_14_ twice. When the sample is inserted into the structure, four major peaks are detected in the waveform. Deconvolving that time-domain waveform identifies the arrival time of the four peaks *t*_1_, *t*_2_, *t*_3_ and *t*_4_ (**Fig *2***B). The distances *L*_12_ and *L*_34_, which are calculated from time-of-flight measurements of the pulses, can be subtracted from *L*_14_ to determine the sample thickness *L* independent of the sample refractive index:

$$L=L_{14}-L_{12}-L_{34}=\frac{c_{o}}{2}[\left(t_{4}{}^{o}-t_{1}{}^{o})-(t_{2}-t_{1})-(t_{4}-t_{3}\right)].$$ (7)

The real refractive index at the peak of the spectral intensity (nominally 0.2 THz) is calculated by

$$n_{r}=\frac{c_{o}(t_{3}-t_{2})}{2L}.$$ (8)

The advantage of using this methodology for measuring the refractive index is that the measurement of the thickness *L* using the propagation time of terahertz pulses is much more accurate than by using calipers.

Using a standard propagation of errors analysis, the relative uncertainty in the real refractive index can be derived from Eqs (7) and (8) to be

$${\left(\frac{\sigma_{n_{r}}}{n_{r}}\right)}^{2}={\left(\frac{\sigma_{t_{23}}}{t_{23}}\right)}^{2}+\frac{{\left(\sigma_{t_{{}_{14}}^{o}}\right)}^{2}+{\left(\sigma_{t_{12}^{}}\right)}^{2}+{\left(\sigma_{t_{{}_{34}}^{}}\right)}^{2}}{{\left(t_{14}^{o}-t_{12}-t_{34}\right)}^{2}}$$ (9)

where the times *t*_*mn*_ is short hand notation for the time difference *t*_*m*_−*t*_*n*_. The mean values for the time delays $t_{14}^{o}$, *t*_12_, *t*_23_, and *t*_34_ are determined from 10000 averages of the deconvolved terahertz time-domain waveforms. Based on the standard deviation of repeated 10000 average measurements, the uncertainty in the various time-delays is typically $\sigma_{t_{23}}$ ~ 0.02 ps. As an example, Eqs (7)–(9) yield a refractive index and uncertainty of *n*_*r*_ = 1.600±0.003 for Baltic amber. The corresponding relative error in the sample thickness is 0.15%.

### Amber samples

To complete imaging and characterization objectives, amber samples were characterized from a total of ten deposits varying in age, suggested botanical origin, and locality (**Table *1***). Samples were trimmed with polished faces, which were close to parallel.

**Table 1:** Summary of amber deposits sampled.

Deposit	Deposit Locality	Deposit Age*	Suggested Botanical Source	Resin Class	Reference
Dominican Amber	Santiago, Dominican Republic	Miocene (16–18 Ma)	Fabaceae	Ic	[9]
Mexican “Chiapas” Amber	Chiapas, Mexico	Miocene (16–23 Ma)	Fabaceae	Ic	[35–37]
Baltic Amber	Baltic Sea	Eocene (34–48 Ma)	Sciadopityaceae	Ia	[9,14]
Arkansas Amber	Claiborne Formation, Arkansas, USA	Eocene (50–56 Ma)	Dipterocarpaceae	II	[38,39]
Wyoming Amber	Hanna Basin, Wyoming, USA	Cretaceous-Paleocene (51–71 Ma)	? Taxodiaceae (or Pinaceae)	?	[40]
Cambay Amber	Gujarat, India	Paleocene-Eocene (52 Ma)	Dipterocarpaceae	II	[15]
New Jersey “Raritan” Amber	New Jersey, USA	Upper Cretaceous (90–94 Ma)	Cupressaceae	Ib	[41]
Burmese Amber	Kachin State, Myanmar	Cretaceous (99 Ma)	Araucariaceae	Ib	[42,43]
Spanish Amber	Las Penosas Formation, Spain	Cretaceous (101–113 Ma)	Araucariaceae	?	[44]
Lebanese Amber	Bcharre, Lebanon	Early Cretaceous (125–129 Ma)	Araucariaceae (or Cheirolepidiaceae)	Ib	[45,46]

Age of stratigraphic layer or reported estimate from reference.

### Data visualization

Scatterplots and color permittivity plots were generated in R v.3.6.1 [47] using the package *ggplot2* [48] and the visualization package *viridris* [49].

## Results and discussion

### Terahertz imaging of inclusions and defects

Research on amber is primarily centered around inclusions such as arthropoods and vertebrates [13,50]. Biological inclusions inform understanding of evolutionary processes and the temporal context for key phenotypic features (e.g. Sherratt et al. [51]). Other technologies, such as X-ray computed tomography, have led to unexpected discoveries, such as the identification of potential metal sequestration in the earliest ants [52].

**Fig 3:**
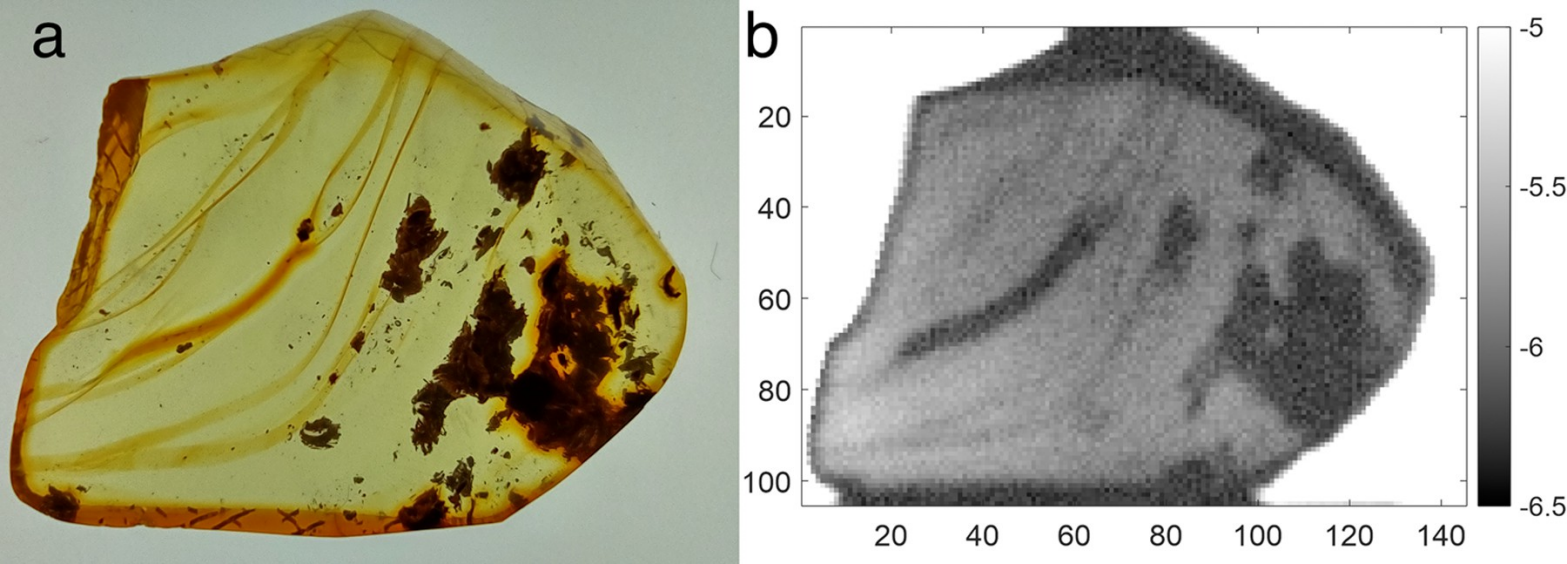
Imaging amber and inclusions. (a) Visible image of a sample of Baltic Amber with flow lines and inclusions. (b) Terahertz transmission image (1.5–2 THz) through the same sample on a natural logarithmic scale (scale value equals ln(T)). For the Terahertz image (29 by 21mm), the pixel resolution is 0.2 mm. For each pixel, twelve waveforms are averaged. The speed of imaging is 5 mm/s. The entire image took approximately 10 min to acquire.

In **Fig *3***, the feasibility of detecting and identifying organic/ inorganic inclusions flow lines, and cracks using terahertz spectroscopy is demonstrated. The image is generated by calculating the magnitude of the terahertz electric field transmission |*E*_*S*_(*ω*)/*E*_*R*_(*ω*)| in the frequency band from 1.5–2 THz. Note that there is sufficient sensitivity and contrast in the image to identify resin flows as well as inorganic and organic inclusions. The contrast in the terahertz image results from increased scattering or absorption by inclusions.

### Extraction of terahertz optical properties–time-of-flight

**Fig 4:**
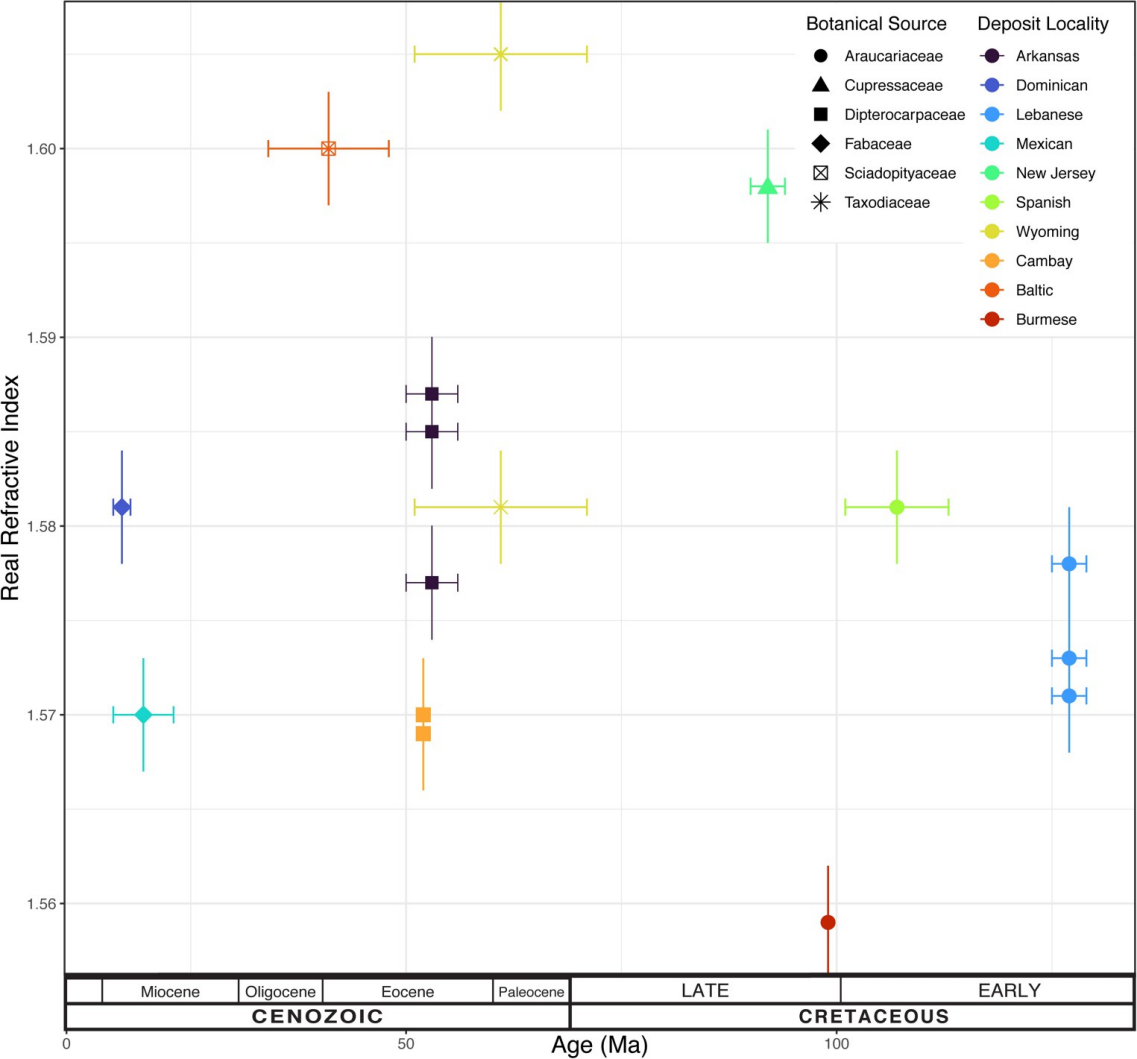
Real refractive index as measured by time-of-flight versus sample age. Botanical source and locality indicated by point shapes and colors, respectively. Horizontal bars denote age ranges noted in Table 1, vertical bars depict uncertainty of the refractive index measurements at ±0.003.

Table 2 lists the various amber samples as well as time-of-flight measured sample thickness and real refractive indices. Data are plotted in **Fig *4*** and denoted by groupings of the suggested botanical source and sample location. Both Arkansas and Cambay amber is suggested derive from a source plant within the family Dipterocarpaceae. Note that the Arkansas samples consistently exhibit a larger real refractive index compared to samples from Cambay. The Arkansas samples are all from the same locality, while samples Ark1_B and Ark3_C exhibit nearly identical refractive indices, sample Ark3_B exhibits a slightly lower value. We attribute this lower value to the presence of ‘striations’ in Ark1_B and Ark3_C. These optically opaque striations may be due to mineral or plant matter inclusions in the amber which would increase the measured refractive index. The Ark3_B sample, which exhibits the lowest real refractive index of the Arkansas samples is much more transparent in the visible range compared to the other Arkansas samples.

**Table 2:** Table of amber sample locality, thickness, and real refractive index values as measured by time-of-flight.

Sample	Deposit	Thickness (mm)	Real Index
AMNH-Bal_mex1_b*	Mexican amber	11.5	1.570
AMNH-Bal_DR1_A*	Dominican amber	8.06	1.581
AMNH-BAL_BA-A*	Baltic amber	3.91	1.600
AMNH-Bal_Ark1_B	Arkansas amber	8.79	1.587
AMNH-Bal_Ark3_B*	Arkansas amber	8.05	1.577
AMNH-Bal_Ark3_C	Arkansas amber	8.08	1.585
AMNH-Bal_Cam1-B	Cambay Amber	9.34	1.569
AMNH-Bal_Cam1-c*	Cambay Amber	10.1	1.570
AMNH-Bal_WY01_A*	Wyoming amber	9.79	1.581
AMNH-Bal_WY01_B	Wyoming amber	9.89	1.605
AMNH-Bal_NJ1_c*	Raritan amber	8.48	1.598
AMNH-Bal_BU-0618*	Burmese amber	1.84	1.559
AMNH-Bal_SPA1_A*	Spanish amber	10.8	1.581
AMNH-Bal_Leb1_B	Lebanese amber	7.55	1.578
AMNH-Bal_Leb1_c*	Lebanese amber	9.60	1.573
AMNH-Bal_Leb1_D*	Lebanese amber	11.0	1.571

The real refractive index value is nominally measured at approximately 0.2 THz light which is the peak of the terahertz transmission in the frequency domain. The uncertainty for the refractive index measurements is about ±0.003 while the relative error for the thickness measurements is about 0.15%. Samples denoted with an asterisk* indicate samples which exhibited sufficient clarity (lack of inclusions) to be characterized spectroscopically.

The Cambay amber samples Cam1_C and Cam1_B, which are from same deposit, have nearly identical refractive indices. While there seems to be good reproducibility among dipterocarp samples from the same deposits, overall, there is a measurable spread (i.e. significantly larger than the estimated error) in their real refractive index values, which may result from their differences in age, geological conditions of formation, or variation in resin chemistry at lower taxonomic units.

The Lebanese amber samples (suggested to be derived from Cheirolepidiaceae or Araucariaceae) exhibit nearly identical real refractive index values. These samples are from the same locality. All are visibly clear and exhibit essentially the same real refractive index within the experimental uncertainty. Comparing the clear Lebanese samples (Leb1_C and Leb1_D) to the clear Spanish sample show a small but measurable difference in the measured real refractive index even though the ages of the samples are similar and the botanical species of these samples may be close relatives (i.e. Araucariaceae).

The two Wyoming samples are from the same locality, however, their real refractive indices are significantly different. The WY01_A sample is visibly transparent and nearly inclusion free while the WY01_B sample is turbid and contains numerous presumably inorganic inclusions mixed in with amber which could be increasing the refractive index of the material. While the samples are from the same deposit, the material composition of the two samples is significantly different resulting in a measurable difference in the refractive indices.

The general conclusions from the time-of-flight measurements are:

1. For samples without inclusions from the same deposit, the measured refractive indices of ‘clear’ amber are very nearly equal.
2. The introduction of inclusions or general turbidity into the amber generally will cause the refractive index to increase relative to ‘clear’ amber samples from the same locality.
3. From the data of Fig 4, there is no obvious trend that relates the sample age or botanical source (at a higher taxonomic level) to the real refractive index.

### Extraction of terahertz optical properties–spectroscopy

It is well-known in terahertz spectroscopy that the presence of inclusions such as cracks, voids, and organic matter will scatter or absorb terahertz radiation thereby distorting the spectral shape of the transmission. (This is the contrast mechanism which gives rise to the terahertz image in **Fig *3***). In order to avoid this anomaly in the spectroscopic data, only samples in **Table *2*** (denoted with an asterisk*) which are sufficiently ‘clear’ are characterized by terahertz spectroscopy. Since the samples are relatively thin (<12mm), inspection of visible light transmission through the samples provides an easy methodology for quickly selecting samples with few or no inclusions.

For all data presented in this section, the lower frequency bound of the data is determined by two effects: (a) signal-to-noise ratio of detected terahertz electric field and (b) sample size relative to the beam size. Terahertz time-domain instruments typically have very little spectral power below about 0.1 THz. However, a further limit to the lowest detectable frequency is the sample size relative to the diameter of the focused beam on the sample. For the work presented in this paper, the terahertz beam is focused onto the sample with a 3-inch focal length lens. Using a simple Gaussian beam analysis, the spot size of the beam is frequency dependent and increases for lower frequencies. For the 3-inch focal length used in these experiments, the expected beam diameter at 0.2 THz which contains 99% of the terahertz power is ~7mm. For sample sizes smaller than the beam diameter, a portion of the power is detected which does not pass through the sample. Consequently, the terahertz transmission spectrum can be distorted for sufficiently small samples. Inserting a fixed aperture just in front of the sample ensures that the detected beam passes through the sample, but at the expense of reducing the detected power at the lower frequency range. The upper limit of spectral measurements is determined by the signal-to-noise ratio. In the absence of any sample, the terahertz spectral power decreases exponentially with increasing frequency. The terahertz system used in this study is capable of detecting signals up to about 2.5 THz. However, the attenuation of power by the amber increases with frequency as well as with sample thickness. Typically, only the spectral data which is free from distortions due to finite sample size and within the signal-to-noise limits at the higher terahertz frequencies is plotted.

**Fig 5:**
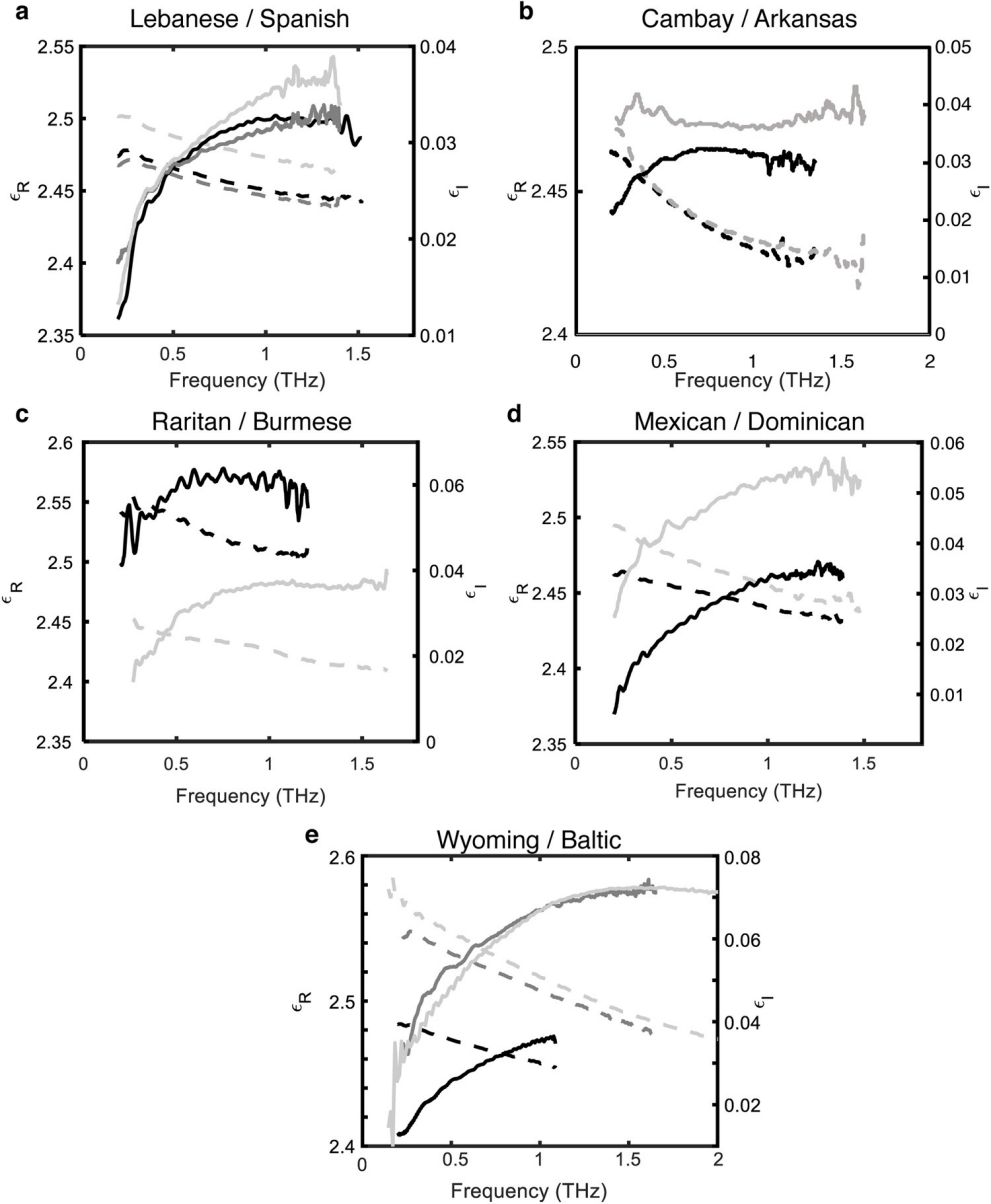
Comparative real and imaginary permittivity of amber localities. Real and imaginary parts of the permittivity are calculated using Eqs (3)–(5). Real permittivity depicted on left axes with dashed lines; imaginary permittivity depicted on right axes with solid lines. (a) Lebanese and Spanish amber: Spectral response for Leb1_C (black lines), Leb1_D (dark gray lines), and Spa1_A (light gray lines) amber samples. (b) Cambay and Arkansas amber: Cam1-C (black) and Ark3-B (gray) dipterocarp samples. (c) Raritan and Burmese amber: NJ1_C (black lines) and BU-0618 (light gray lines) Cretaceous samples. (d) Mexican and Dominican amber: Mex1_B (black lines) and DR1_A (gray lines) amber samples. (e) Wyoming and Baltic amber: WY01_A (black lines) and BA-A (dark gray lines) amber samples. The spectral data of Sasaki et al. Baltic data [28] are shown in light gray.

As an initial step, it was verified experimentally that two samples of inclusion-free amber from the same deposit produced nearly the same frequency dependent complex permittivity. For example, **Fig *5*** shows the extracted frequency dependent real and imaginary parts of the permittivity by analyzing the transmission data using Eqs (3)–(5). The spectral analysis of Eqs (3)–(5) does not take into account Fabry-Perot interference fringes between the front and back surfaces of the nearly parallel sample faces. To remove this effect from the data, the spectral data is smoothed using a 10–15 point moving average. Note the good reproducibility of the data for Lebanese amber over the measured frequency range. The Spanish amber is also plotted for comparison since its botanical source may be the same as the Lebanese amber. Note that the spectral shapes of the real permittivity are very similar, but the Spanish amber exhibits a larger real permittivity. While the imaginary permittivity show similar spectral peaks near 1.3 THz, the Spanish amber exhibits a larger peak imaginary permittivity. In the dielectric relaxation model of Eq (2), the frequency peak of the imaginary permittivity which depends on *τ*, *σ*_*CC*_, and *σ*_*CD*_ can serve as a measure of the time-scale for dielectric relaxation. The similar frequency location for the spectra peaks for Spanish and Lebanese amber may suggest a common botanical source. Indeed, species within the present-day relict conifer genus *Agathis* have been proposed as potential source resins for both Spanish and Lebanese amber, although numerous other taxa have also been suggested based on fossilized plant associations and amber chemistry (Seyfullah et al. 2018).

In addition to above qualitative analysis, the imaginary permittivity curves of **Fig *5*** were fit to Eq (2) to extract best fit parameters for the Haavriliak-Negami, Cole-Davidson, Cole-Cole, and Debye models. The extracted model parameters are given in the S1 Data.

The quantitative analysis of the fitting parameters appears to highlight an inconsistency in the Lebanese amber: the two curves which qualitatively look very similar exhibit significantly different fitting parameters except for a Debye model fit. We attribute this inconsistency to the limited spectral range (0.2–1.4 THz) of the fit. The location of the spectral peak in the imaginary permittivity determines the dielectric relaxation time *τ*. Since the spectral responses of the Lebanese and Spanish amber in **Fig *5***C appear to peak near the upper limit of the spectral data, the parameter *τ* may not be accurately determined. The same argument can be made concerning the Burmese, Mexican, Dominican, Wyoming, and Baltic amber samples in **Fig *5***: only the Cambay and Raritan amber exhibit spectral peaks in the imaginary permittivity at sufficiently low terahertz frequencies to reliably determine the dielectric relaxation time *τ*.

The Dipterocarpaceae samples (Fig 5B) show very similar real permittivity spectra but significant variations in the imaginary permittivity spectra among the Arkansas and Cambay samples. While the Cam1-C sample shows a characteristic spectral shape of *ε*_*I*_, Ark3_B shows a much flatter spectral shape. The spectrally flat *ε*_*I*_ of Ark3_B is most likely an artifact of remaining inclusions in the sample. Fitting of the Arkansas imaginary permittivity data to Eq (2) produces very poor fits with relatively low R^2^ values and large residuals. Even though the sample is the most visibly transparent of the Arkansas amber samples, there are still significant inclusions and internal structure which could be distorting the *ε*_*I*_ spectra.

**Fig *5***C shows a comparison of the spectrally dependent real and imaginary parts of the permittivity for Late Cretaceous samples. The New Jersey sample shows a larger real and imaginary permittivity compared to the Burmese sample. Moreover, the spectral shape of the imaginary permittivity is different for the two samples. The New Jersey sample exhibits a broad peak near 0.75 THz while the Burmese sample show a peak near 1.2 THz suggesting that the time-scales for dielectric relaxation for these two samples are distinct. The distinctly different time-scales are also evident in the Cole-Davidson fit parameters.

Fig 5D shows a comparison of the permittivity spectra for Fabaceae amber from the Dominican Republic and Mexico. While the Dominican amber exhibits a higher *ε*_*I*_, the spectral shapes of the permittivity are very similar between the two samples perhaps suggesting that they are from a similar deposit and experienced similar geological conditions during polymerization of the source resin. Notably, both deposits are known to derive from species of the genus *Hymenaea* in the family Fabaceae [36]. Comparing the extracted parameters for *τ* and *σ*_*CC*_ for the Cole-Cole model suggests similar relaxation times. However, the extracted time constants for the Cole-Davidson model are significantly different for these two samples. This discrepancy may result from an inability to accurately determine, as noted above, the frequency of the imaginary permittivity spectral peak.

Fig 5E shows a comparison of Wyoming and Baltic amber. In addition, the spectral data of Sasaki et. al [28] is shown. The Baltic amber spectral data from the present work qualitatively agrees very well with the previous results of Sasaki over the datasets common spectral range. The extracted best fit parameters for the two Baltic samples using the Cole-Cole model (Supplemental Data 1) are also very similar. However, the best fit parameters for the two Baltic samples using the Cole-Davidson model show significant differences suggesting that spectroscopic data over a wider spectral range is required to determine the best dielectric relaxation model for different amber deposits.

### Evaluation of counterfeit amber

Amber is frequently fabricated because of its commercial value. Synthetic polymers and glasses are sold with modern organisms imbedded and marketed as “fossil amber.” Such forgeries may mislead scientific research as counterfeit material may even be unwittingly donated to museums [53]. To evaluate the efficacy of terahertz spectroscopy in detecting forgeries, we purchased and assessed the relative terahertz permittivity of an obviously human-made counterfeit amber sample: a large scorpion embedded in homogenous, yellow-colored transparent resin described as “Beautiful amber scorpion fossil insects manual polishing.” The forgery matrix is undoubtably composed of synthetic resin with an added resin colorant, although the exact chemical composition is unknown. There remain more sophisticated approaches, including hollowing out genuine amber specimens and placing extant inclusions inside, however we did not assess such forgeries here.

**Fig 6:**
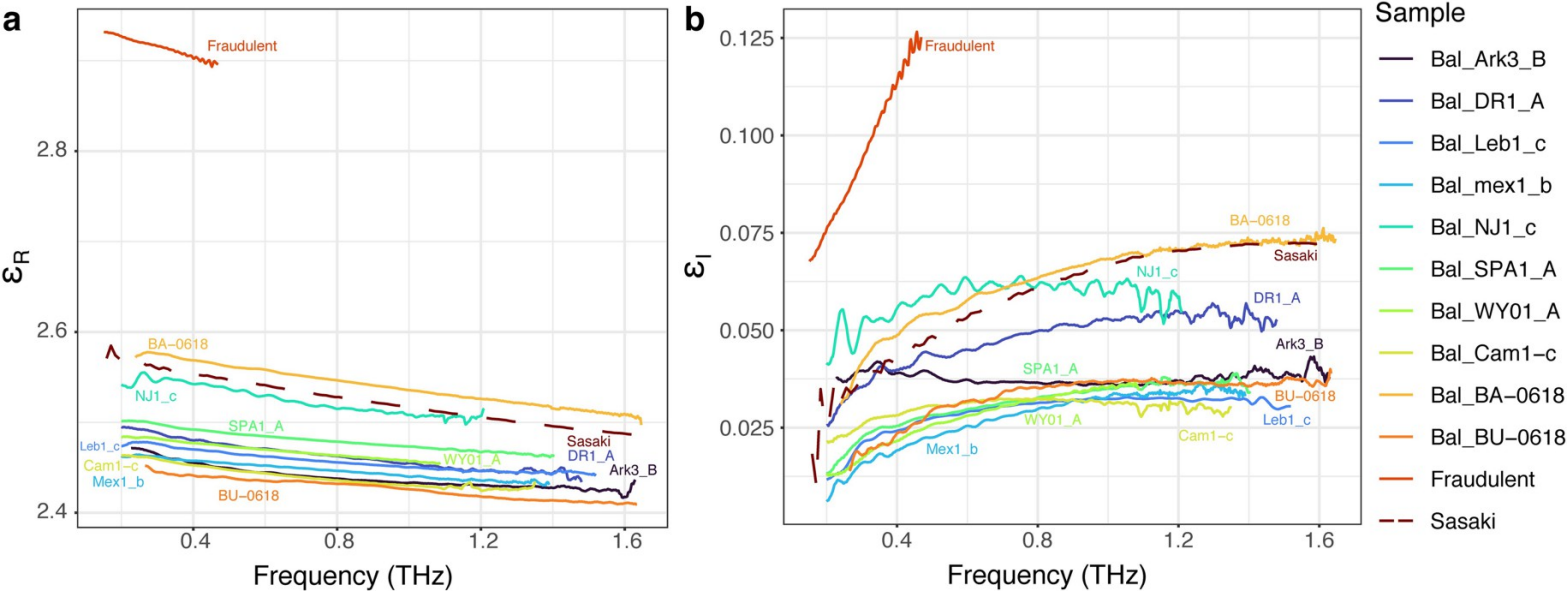
Evaluation of fraudulent synthetic amber relative to amber samples. Extracted real (a) and imaginary (b) permittivity from selected clear amber samples. The previous Baltic Amber results of Sasaki et al. [28] are shown as a dashed line for comparison. The Sasaki et al. [28] data are in close agreement with the spectral response of Baltic Amber measured in the present paper.

Using the same experimental and analytical methodology for amber samples, the complex permittivity of the fraudulent amber was determined. Fig 6 shows the measured real and imaginary permittivity of clear amber samples compared to the counterfeit amber. The spectral range of data for the fake amber is more limited compared to the clear amber samples due to the significantly larger attenuation of the terahertz radiation in fraudulent amber. The data clearly shows that the synthetic resin for this particular fraudulent sample is easily distinguished from real amber based on the measured permittivity. It is likely that some synthetic resins may exhibit both real and imaginary permittivity that is more similar to that of genuine fossil resin. Future comparative assessments of synthetic, optically transparent resins using terahertz spectroscopy may reveal the limits of counterfeit detection using this method.

## Conclusions

We present an initial characterization of amber samples representing a variety of ages, botanical sources, and geographic locations with terahertz spectroscopy. The broad spectral range of data on Baltic Amber previously published by Sasaki et al. [28] enabled the fitting of the complex permittivity to functional forms of the Haavriliak-Negami equation. Better fits to the experimental data were acquired using a Cole-Davidson model, indicating that Baltic Amber is better described by an asymmetric distribution of dielectric relaxation times. Time-of-flight terahertz measurements through amber demonstrate that, for samples without inclusions from the same deposit, the measured refractive indices of transparent samples are very nearly equal. The introduction of inclusions (including turbidity or minerals mixed in with the amber) into the amber generally will cause the refractive index to increase relative to ‘clear’ amber samples. There is no obvious trend that relates the sample age or botanical source (at a higher taxonomic level) to the real refractive index across samples we assessed. Additional work is needed to generate a large-scale dataset of amber with known chemistries and terahertz transmission.

Using a Fourier transform analysis method to analyze the terahertz data in the frequency domain, comparison of the real and imaginary permittivity spectra shows notable trends. While the spectral shape of the real permittivity is very similar for all clear amber samples, the spectral shape of the imaginary permittivity varies with amber sample indicating that dielectric relaxation time of the amber could depend on a combination of the geographic locale of the amber, botanical source, and local geological parameters. Best fits to the imaginary permittivity suggest that the either the Cole-Cole or Cole-Davidson model of dielectric relaxation better describes the measured spectral permittivity data compared to the Debye model for most amber deposits. However, due to the limited range of spectral data for most of the deposits, it is difficult to further quantify the comparison. Comparison of the measured permittivity of counterfeit amber shows that terahertz spectroscopy can easily distinguish between a fraudulent specimen derived from at least some synthetic resins and amber.

## Supporting information

S1 Data Model summary statistics. (CSV)

S2 Data Raw permittivity data. (XLSX)
